# Research on the treatment of rifampin-susceptible tuberculosis—Time for a new approach

**DOI:** 10.1371/journal.pmed.1004438

**Published:** 2024-07-25

**Authors:** William Burman, Oxana Rucsineanu, C. Robert Horsburgh, James Johnston, Susan E. Dorman, Dick Menzies

**Affiliations:** 1 Public Health Institute at Denver Health and the Department of Medicine, University of Colorado, Denver, Colorado, United States of America; 2 Moldova National Association of Tuberculosis Patients “SMIT” (Society of Moldova against Tuberculosis), Balti, Moldova; Global TB Community Advisory Board, New York, New York, United States of America; 3 Departments of Global Health, Epidemiology, Biostatistics and Medicine, Schools of Public Health and Medicine, Boston University, Boston Massachusetts, United States of America; 4 Division of Respiratory Medicine, University of British Columbia, Vancouver, Canada; 5 Division of Infectious Diseases, Department of Medicine, Medical University of South Carolina, Charleston, South Carolina, United States of America; 6 McGill International TB Centre, Montreal Chest Institute, Research Institute of the McGill University Health Centre, Montreal, Canada

## Abstract

In the Perspective, William Burman and colleagues advocate improving the safety and acceptability of treatment, rather than treatment-shortening, of rifampin-susceptible tuberculosis.

## Brief summary (nonscientific “standfirst”)

*Despite 25 years of clinical trials, there has been only modest progress in improving treatment of rifampin-susceptible tuberculosis. We propose that clinical trials should shift the focus to improving the safety and acceptability of treatment, rather than treatment-shortening. Because adverse events increase the risk of treatment failure and recurrence, a focus on safety and acceptability should result in an improvement in both the patient’s experience of treatment and traditional tuberculosis treatment outcomes*.

There has been renewed activity in clinical trials for tuberculosis treatment over the past 25 years. The greatest success of these randomized trials has been the transformation of treatment for multidrug-resistant tuberculosis to 6-month, all-oral regimens. Tuberculosis preventative therapy has also markedly improved with the development of safer, shorter regimens. There has been modest progress in the treatment of rifampin-susceptible tuberculosis disease, but these new regimens have failed to address the most important aspect of treatment needing improvement: safety (freedom from severe side effects) and acceptability to people experiencing tuberculosis. We believe it is time to take a new approach to clinical trials in this area.

Clinical trials for rifamycin-susceptible tuberculosis in the past 25 years have been largely directed toward the fundamental goal of shortening treatment, by substituting new drugs or increasing doses of drugs in the standard regimen (2 months of rifampin, isoniazid, pyrazinamide, and ethambutol, followed by 4 to 7 months of rifampin and isoniazid). Regimens evaluated in these trials were chosen based on their increased antimycobacterial activity in animal models and on surrogates of treatment outcomes in humans (e.g., 2-month sputum culture conversion). The singular focus on treatment-shortening, while maintaining efficacy, suggests that treatment duration is the most important barrier to completing effective therapy. These trials have mostly used non-inferiority methodology, which implies that the current standard therapy is effective, safe, and well tolerated.

Current tuberculosis treatment is neither safe nor acceptable to patients—bothersome side effects, such as nausea, are common and often result in treatment being changed by providers or abandoned by patients [1–3]. Severe side effects are common, as well. For example, 10% of participants in a recent large trial had grade 3 or higher treatment-related adverse events [4]. Current treatment is very poorly tolerated in populations that are increasingly important in the global tuberculosis pandemic—persons older than 50 years and those with concomitant medical illnesses such as diabetes mellitus and alcohol use disorder. For example, in recent cohort studies, treatment was changed or interrupted in 25% to 45% of adults aged 65 years and older [1,2]. Furthermore, it is notable that the risk of severe hepatoxicity from tuberculosis treatment has more than doubled (from approximately 7% to 16%) from 1999 to 2020 [5], perhaps related to the increasing age of patients with tuberculosis.

Recent Phase 2 clinical trials have prematurely concluded that new regimens are safe based on absence of statistically significant increases in toxicity with experimental regimens compared with the current standard, yet sample sizes in these trials (generally <200 patients per arm) were inadequate to draw conclusions about safety [6]. Furthermore, many Phase 3 treatment-shortening trials underestimated the toxicity and intolerability of both standard and new treatment regimens currently in use by enrolling predominantly young adults [4], a group at lower risk of toxicity. Finally, a critical finding in multiple studies of tuberculosis treatment is that adverse events are associated with poor tuberculosis treatment outcomes (treatment failure, recurrence, or death) [3,7]. For example, in the ReMox trial, participants in the standard therapy arm who had grades 3 to 4 adverse events had a >3-fold higher risk of poor tuberculosis treatment outcomes [7]. The lesson is clear: toxicity from treatment decreases cure. Therefore, to improve treatment completion and increase cure, we need regimens that are *safer and more acceptable* than current therapies [8], not regimens that are merely no worse.

The development of the 4-month rifampin regimen for tuberculosis preventative therapy provides an example of how we think clinical trials should be approached in populations with rifampin-susceptible tuberculosis: an initial small trial showed better treatment completion with 4 months of rifampin compared to 9 months of isoniazid, a subsequent trial showed lower rates of hepatotoxicity, and then a large trial showed non-inferiority in progression to active TB [9]. Focusing on treatment completion through improved acceptability has been critical in the success of short-course, all-oral regimens for rifampin-resistant tuberculosis. The superiority of the all-oral regimens in the TB-PRACTECAL trial resulted from lower rates of modification of assigned study therapy, loss to follow-up, and study withdrawal, rather than lower rates of treatment failure and relapse [10].

Therefore, we propose a new approach to clinical trials of rifampin-susceptible tuberculosis: superiority trials of regimens with drugs chosen based on the likelihood that they will be safer and better tolerated than current therapy. The primary endpoint of tolerability trials should be on-time completion of assigned study therapy without toxicity (Table 1). The US Food and Drug Administration recently emphasized that outcomes that capture the patient’s experience of therapy play a central role in regulatory decision-making [11]. Traditional efficacy outcomes (treatment failure, recurrence, death during treatment, 2-month sputum culture conversion) should be assessed as secondary endpoints during the initial evaluation of new regimens. When regimens with improved safety and acceptability have been identified, subsequent late-phase trials can be powered to compare traditional efficacy outcomes. By focusing on tolerability and safety, we should also enable enrollment of a broader and more representative spectrum of people with tuberculosis. Enrollment of older individuals, those with other chronic illnesses and concomitant medications, people with extrapulmonary involvement, and pregnant/lactating women should be a priority.

**Table 1:** Comparison of the current and proposed approaches for treatment trials of rifampin-susceptible tuberculosis.

	Current approach	Proposed approach
Primary objective	Increase treatment completion by shortening treatment duration	Increase treatment completion by improving safety and acceptability
Regimen selection	Antimycobacterial potency, as assessed in animal models, results of Phase 2 trials with microbiological surrogates (e.g., 2-month sputum culture conversion)	Results of Phase 2 trials of safety and acceptability, antimycobacterial activity as assessed in animal models and surrogates in Phase 2 trials
Primary endpoint	Composite of treatment failure, recurrence*, or death	On-time completion of assigned study therapy in trials that include patient decision to change, modify, or stop therapy
Key secondary endpoints	Grade 3 or 4 adverse events, 2-month sputum culture conversion	TB treatment failure, recurrence, or death, patient-reported outcomes, adverse events over time, grade 3 or 4 adverse events
Key inclusion criteria	Culture positive pulmonary TB†	Treatment for proven or presumed rifamycin-susceptible TB (pulmonary or extra-pulmonary) to include persons of all ages and comorbidities

*Some trials excluded participants in which the isolate from the time of recurrence had a different DNA fingerprint from the initial isolate (presumed re-infection).

†Requiring sputum culture positivity decreases the ability to include populations that often have negative sputum cultures and/or extrapulmonary disease (children, persons with concomitant medical conditions).

Another key element in focusing on tolerability and safety must be assessment of patient-reported outcomes and the analysis of less severe adverse events (i.e., not just grade 3/4 events). Advocates recently articulated that “… with a myopic focus on treatment duration, researchers and clinicians are missing opportunities to address other aspects of treatment that are challenging for people with TB” [12]. Finally, we need trials of tuberculosis interventions to minimize and manage both common and severe side effects of tuberculosis treatment (e.g., concomitant use of anti-emetic drugs or hepatoprotective agents). The oncology field has shown that a focus on managing side effects can improve treatment outcomes.

The long-term goal of research on treatment of rifampin-susceptible tuberculosis is the development of regimens that are acceptable and safe, highly effective, short, and can be used by all people who have the disease. The almost exclusive focus of current research on antimycobacterial potency and treatment shortening is flawed. Each of the drugs in the current regimen has substantial limitations, underscoring the need for basic and translational research to identify new drugs. These new drugs and regimens should be evaluated in clinical trials that prioritize safety and acceptability. While awaiting new drugs, clinical trials can evaluate whether changes in currently available drugs could improve the safety and acceptability of tuberculosis regimens, for example, elimination of pyrazinamide, substitution of a fluoroquinolone for isoniazid, and/or substitution of rifapentine for rifampin [6]. Finally, we need to hear more from people affected by tuberculosis. By focusing on outcomes prioritized by patients, we are likely to identify regimens with better outcomes.
